# Benefits of Diabetes Self-Management for Health Plan Members: A 6-Month Translation Study

**DOI:** 10.2196/jmir.5568

**Published:** 2016-06-24

**Authors:** Kate Lorig, Philip L Ritter, Ralph M Turner, Kathleen English, Diana D Laurent, Jay Greenberg

**Affiliations:** ^1^ Stanford School of Medicine Stanford University Palo Alto, CA United States; ^2^ HealthCore Wilminton, DE United States; ^3^ Anthem, Inc Indianapolis, IN United States; ^4^ NCOA Services, LLC National Council on Aging Arlington, VA United States

**Keywords:** patient education, self-management, type 2 diabetes, translation and dissemination

## Abstract

**Background:**

Diabetes self-management education has been shown to be effective in controlled trials. However, few programs that meet American Association of Diabetes Educators standards have been translated into widespread practice.

**Objective:**

This study examined the translation of the evidence-based Better Choices, Better Health-Diabetes program in both Internet and face-to-face versions.

**Methods:**

We administered the Internet program nationally in the United States (n=1010). We conducted face-to-face workshops in Atlanta, Georgia; Indianapolis, Indiana; and St. Louis, Missouri (n=232). Self-report questionnaires collected health indicator, health behavior, and health care utilization measures. Questionnaires were administered on the Web or by mail. We determined hemoglobin A_1c_ (HbA_1c_) from blood samples collected via mailed kits. Paired *t* tests determined whether changes between baseline and 6 months differed significantly from no change. Subgroup analyses determined whether participants with specific conditions benefited (high HbA_1c_, depression, hypoglycemia, nonadherence to medication taking, and no aerobic exercise). We calculated the percentage of participants with improvements of at least 0.4 effect size in at least one of the 5 above measures.

**Results:**

Of the 1242 participants, 884 provided 6-month follow-up questionnaires. There were statistically significant improvements in 6 of 7 health indicators (including HbA_1c_) and in 7 of 7 behaviors. For each of the 5 conditions, there were significant improvements among those with the condition (effect sizes 0.59–1.1). A total of 662 (75.0%) of study participants improved at least 0.4 effect size in at least one criterion, and 327 (37.1%) improved in 2 or more.

**Conclusions:**

The Diabetes Self-Management Program, offered in two modes, was successfully disseminated to a heterogeneous national population of members of either insured or administered health plans. Participants had small but significant benefits in multiple measures. The program appears effective in improving diabetes management.

## Introduction

### Background

Type 2 diabetes is a growing problem. Not only is the prevalence increasing, but also there are increased costs and decreased quality of life. Patients, health care providers, families, employers, and insurers all share this burden. The US government, in Healthy People 2020, set national objectives for improvements in diabetes care and outcomes [1].

Diabetes self-management education has been shown to be effective in improving health behaviors and in some cases lowering hemoglobin A_1c_ (HbA_1c_). However, <7% of privately insured persons receive formal education in the year after diagnosis [2] and <60% report having attended a diabetes class [1]. The American Association of Diabetes Educators has set standards for diabetes self-management education [3,4]. Few programs, however, that meet these standards have been translated into widespread practice and shown to be effective. One reason for this is that most programs are not easily translatable: they do not have (1) standardized curricula that are manualized in such a way that minute-to-minute details are given of exact content to be presented, as well as the processes by which this content is offered, (2) standardized training programs, and (3) manuals for fidelity standards. All 3 components are necessary for effective replication.

The Administration for Community Living (US Department of Health and Human Services) and the Evidence-Based Leadership Council have varied in their definitions of evidence-based programs [5,6]. However, key to both definitions are that the program must have been shown to be effective and that it have one or more peer reviewed publications. In addition, there must be infrastructure for translation, and the program must have been replicated in sites other than where it originated. The National Diabetes Education Program does not define evidence-based diabetes education but does suggest using programs that meet the National Standards for Diabetes Self-Management Education [3]. None of these definitions include the notion that the intervention must be shown to be effective outside of its home site.

### A National Translation Study

In this paper, we present the national translation of an evidence-based diabetes education program, The Stanford Diabetes Self-Management Program, also known as Better Choices, Better Health-Diabetes (BCBH-D) [7-9], given via the Internet or in small face-to-face community groups.

Recent systematic reviews have found that both community and Internet interventions have been effective in reducing HbA_1c_ and improving quality of life for people with type 2 diabetes [10-14]. These programs have nearly always been offered in the context of a controlled study. In that setting there are often extensive inclusion and exclusion criteria, which may bias the results when applied to the general diabetes population. This is especially true for HbA_1c_, where the inclusion criterion is seldom <7 (considered to be controlled diabetes) and often as high as 9. For example, in Zhang et al’s meta-analysis of 20 studies using peer support for diabetes, 12 studies had a mean baseline HbA_1c_ of ≥8 [14]. There is limited information about the effectiveness of specific programs when translated to widespread use and offered to large populations by non-health care providers [15,16].

For this translation study, the US National Council on Aging led a collaboration of 5 organizations. It also provided the platform for offering the Internet workshops. Anthem, Inc (Indianapolis, IN, USA) -affiliated health plans recruited participants from their members. The Young Men’s Christian Association of the United States of America in Atlanta, Georgia, and OASIS Institute in St. Louis, Missouri, and Indianapolis, Indiana, offered community programs. The Stanford Patient Education Research Center (Stanford University School of Medicine, Stanford, CA, USA) collected data and provided data analysis. HealthCore (Wilmington, DE, USA), a subsidiary of Anthem, Inc, contributed to the design and data analysis. The team members met for months prior to the beginning of the study to decide on study design, outcome variables, and logistics. They continued to meet at least monthly for the duration of the intervention. The study was approved by the Stanford University Institutional Review Board and New England Independent Review Board.

We hypothesized that over 6 months, people with diabetes participating in the program would demonstrate (1) a reduction in HbA_1c_, (2) a reduction in symptoms (hypoglycemic symptoms and depression), (3) increases in healthful behaviors (exercise, communication with physicians, and medication adherence), (4) increases in receiving recommended tests (eye, foot, cholesterol, and kidney examinations). We also hypothesized (5) that program effectiveness would be independent of mode of delivery, (6) that participants with baseline HbA_1c_ ≥9.0, 8-item Patient Health Questionnaire (PHQ-8) depression ≥10.0, having 2 or more hypoglycemia symptoms, medication nonadherence, or no aerobic exercise would have clinically significant improvements in the variables of interest, and (7) that most of the participants would have a moderate effect size (0.4) improvement in either reducing HbA_1c_, reducing depression, increasing medication adherence, reducing hypoglycemic symptoms, or increasing exercise.

In addition, we wanted to explore the potential of BCBH-D to meet some of the Healthy People 2020 objectives: decrease the proportion of people with diabetes with HbA_1c_ >9, and increase the proportion of people with diabetes who (1) have an annual foot examination, (2) have an annual urinary microalbumin measurement, and (3) receive formal diabetes education.

## Methods

### Intervention

We chose BCBH-D because it was developed for people with type 2 diabetes in real-world settings. We built both programs (face-to-face and Internet) on extensive patient input, as well as assistance from certified diabetes educators. Both programs have been shown to be effective in previous randomized trials [7,9], have the same content, and are taught in an interactive manner designed to enhance self-efficacy [17]. Both have a duration of 6 weeks, 2 peer facilitators, and standardized facilitator training. Participants receive the same book, *Living a Healthy Life with Chronic Conditions* [18], which contains program content and chapters on other chronic conditions. The face-to-face program has detailed facilitator, administrative, and fidelity manuals [19], while the Internet program has an administrative manual for facilitators.

The BCBH-D Internet program is a password-protected, interactive, Web-based program. The user interface consists of 3 major sections. (1) The Learning Center offers 20–30 pages of didactic and interactive content each week. In addition to reading content within the Learning Center, participants make weekly action plans, give feedback on the plan from the previous week, and answer a question such as “What problems do you have with your diabetes?” The action plan, feedback, and questions populate the Discussion Center. (2) The Discussion Center contains 4 interactive bulletin boards: problem solving, action planning, difficult emotions, and celebrations. Participants can post to any of these boards and respond to posts at any time. (3) My Tools is a series of tools, such as a medication log, food diary, exercise diary, and links to other websites that can be used as participants wish.

The Internet facilitators are trained over the Web by first participating in a workshop, followed by Web-based training and then cofacilitating the workshop with an experienced facilitator. Certified diabetes educators are available to facilitators to answer questions as necessary. All interactions between facilitators and participants take place on the Web. Facilitators assist participants with the program. They model action planning and problem solving, and offer encouragement by posting to the discussion boards. Facilitators monitor the daily posts of all participants and report inappropriate posts to program administrators. They also have access to a certified diabetes educator. Unlike in the community program, in the Internet program facilitators do not deliver content, as this is scripted on the Web.

For the Internet program, 25–30 participants log on at least 3 times a week and participate in weekly activities, including reading content, posting an action plan, and interacting on the discussion boards. Any problem a participant wishes to discuss can be posted in the Discussion Center and responded to by other participants. The Internet program mirrors the original community program, except that it does not require real-time attendance. Participants can log in any time, have no face-to-face interaction, and may return to past weeks’ material [7,9].

The community workshops meet for 2.5 hours a week; 10–15 people attend and may bring a friend or family member. This is longer than the 10 hours usually covered by insurance participant’s health plan benefits. The length was determined by the amount of material to be covered, as well as past work that indicated that shorter programs were less effective [20]. In a recent review, Pillay and colleagues also noted that programs with ≤10 hours of contact had limited benefit in improving glycemic control [21].

Both the community and Internet BCBH-D content meets the diabetes self-management education recommendations for diabetes self-management education and support [3]. More than 20 organizations have received American Association of Diabetes Educators certification for the face-to-face program. Although the content and processes are very similar, there is no means for certifying or recognizing Web-based programs. Both interventions have been widely translated into practice. More than 50,000 people in 39 states in the United States have taken the community program and more than 2000 have participated via the Internet. The community program is also used in 14 countries outside the United States. This broad translation is possible because of program and training standardization, because of the original evidence base of effectiveness, and because program delivery meets the needs of community and health care organizations.

### Recruitment

Because we wanted to replicate real-world settings, we stipulated few inclusion (have type 2 diabetes and be covered by an Anthem plan) or exclusion criteria (currently pregnant or had chemotherapy or radiation treatment for cancer in the past year). There were no inclusion criteria based on level of symptom severity, nor was having had cancer by itself an exclusion criterion.

We recruited Internet participants by email or announcements from their employers or emails directly from an Anthem plan. A small percentage of participants were referred by family, friends, or physicians. Participants in both commercial and Medicare Advantage health insurance programs were eligible. Potential study participants went to the recruitment website, completed a screening form, and, if they met study requirements, completed an informed consent and baseline questionnaire.

We recruited face-to-face participants through mailings, flyers in workplaces, case managers, and automated telephone calls. They were asked to call a local number for details about the workshop, screened for study eligibility, and registered. Programs were available in Atlanta, Indianapolis, and St. Louis. A small percentage of the community participants were not covered by an Anthem plan. All other screening criteria were the same for Internet and community participants.

Participants enrolled between October 2013 and October 2014.

### Data Collection

We collected data at baseline, 6 months, and 12 months. This paper reports only on the 6-month results. Internet participants completed consent forms and all questionnaires on the Web. Face-to-face participants completed informed consent forms and baseline questionnaires within a week of their first session. Follow-up questionnaires were sent by mail.

We asked potential participants to supply a capillary sample of blood for HbA_1c_ testing. If willing, they were sent a CoreMedica home test kit (CoreMedica Laboratories, Lees Summit, MO, USA). These were returned to the investigators, bar coded to avoid disclosing private information, and then sent to CoreMedica, a Clinical Laboratory Improvement Amendments-certified laboratory [22]. Participants and their physicians were sent the results. Participants were not required to consent to blood testing, nor were they disqualified if they did not return their tests. Because CoreMedica recalibrated its measurements in June 2014, increasing all values by roughly 0.4, all measures prior to June were adjusted upward by 0.4.

### Measures

We chose measures to be of interest to patients, providers, and the health care system. We gathered all data except HbA_1c_ from validated self-report questionnaires. Demographic variables included age, sex, race, ethnicity (non-Hispanic white), education, and marital status. In addition, we recorded other diseases (asthma, chronic obstructive pulmonary disease, other lung disease, hypertension, heart disease, renal disease, arthritis, cancer, depression or other mental condition). Outcome measures, described below and in full detail elsewhere, fell into two broad categories: health indicators and health behaviors.

All health indicators have been validated in previous studies. A higher score indicates greater symptoms or worse health. Self-rated health consists of a single item from the National Health and Nutrition Examination Survey (range 1–5) [23]. The PHQ-8 depression scale is calculated as the sum of 8 items, with a range of 0–24 [24]. The illness intrusiveness scale consists of 13 items that measure how much a participant’s illness interferes with different aspects of life [25]. Each item ranges from 1 (not very much) to 7 (very much interference). The hypoglycemic symptoms scale was developed by Piette [26]. It is the mean of 7 yes–no questions regarding the presence of different symptoms, with a range of 0–7 symptoms. Fatigue and sleep are each single-item visual numeric scales ranging from 0 (no fatigue or no sleep problems) to 10 (severe fatigue or very big problem sleeping) [27].

The health behaviors measures have also been validated elsewhere. A 3-item scale measured communication with physicians. It used a 6-point scale (from never to always) to measure how often the participant prepared a list of questions for the physician related to the illness [28]. We assessed minutes of aerobic exercise per week by asking about 5 types of aerobic exercise [28]. The Morisky Medication Adherence Scale consists of 4 yes–no items asking about taking medication [29]. The scale has a range of 0–4, with a higher value indicating less adherence. We also asked participants if they had eye, foot, cholesterol, and kidney examinations in the past 6 months and past 12 months.

### Data Analysis

Translation studies, because of study population heterogeneity, present unique methodological challenges. In many diabetes studies, participants are chosen based on having difficulty with the outcome of interest. Thus, participants enter into studies because of high HbA_1c_, depression, hypoglycemia, or being nonadherent. In this study, no such screening occurred. This results in greater heterogeneity, as not all participants have the same problems and some may have none of the problems. Consequently, we conducted two types of analyses. The first, or classic, analyses determined the changes, significance, and effect sizes for the population as a whole. The second, or subset, analyses examined only that portion of the study population that demonstrated problems in the variable of interest, that is, high HbA_1c_ or low adherence to taking medications. A third analysis sought to reconcile these two by examining the percentage of the total population who achieved a moderate benefit (0.4 effect size) in at least one of the variables of interest.

Univariate statistics described demographic characteristics. Independent sample *t* tests compared demographic and baseline outcome variables between those who did not complete 6-month follow-up questionnaires and those who completed them. Paired *t* tests examined changes between baseline and 6 months and whether these differed significantly from a null hypothesis of zero change (hypotheses 1–4).

For participants who had had no examinations (eye, foot, cholesterol, or kidney) in the year prior to entry, we calculated the percentage who had examinations in the 6 months following baseline (hypothesis 4).

To determine effectiveness by mode of delivery (hypothesis 5), we used independent sample *t* tests to compare change scores between Internet and face-to-face participants. We also used analyses of variance to compare differences among the 3 community locations.

We conducted subgroup analyses for participants with specific indicators (hypothesis 6). These were HbA_1c_ >9; clinical depression (PHQ-8 of ≥10 [24]); at least two symptoms of hypoglycemia; low medication adherence; and no exercise at baseline. For each measure, we report the mean change of the group and the percentage who no longer had the negative indication.

To determine whether a large portion of the participants benefited on at least one important outcome, we calculated the percentage who had an improvement of at least 0.4 effect size (change score divided by baseline standard deviation) in at least one of the 5 criterial measures (hypothesis 7). We also calculated the mean number of improvements (out of 5) and examined how this varied by the number of the 5 specific indicators each participant had.

## Results

### Participants and Baseline Demographics

**Figure 1:**
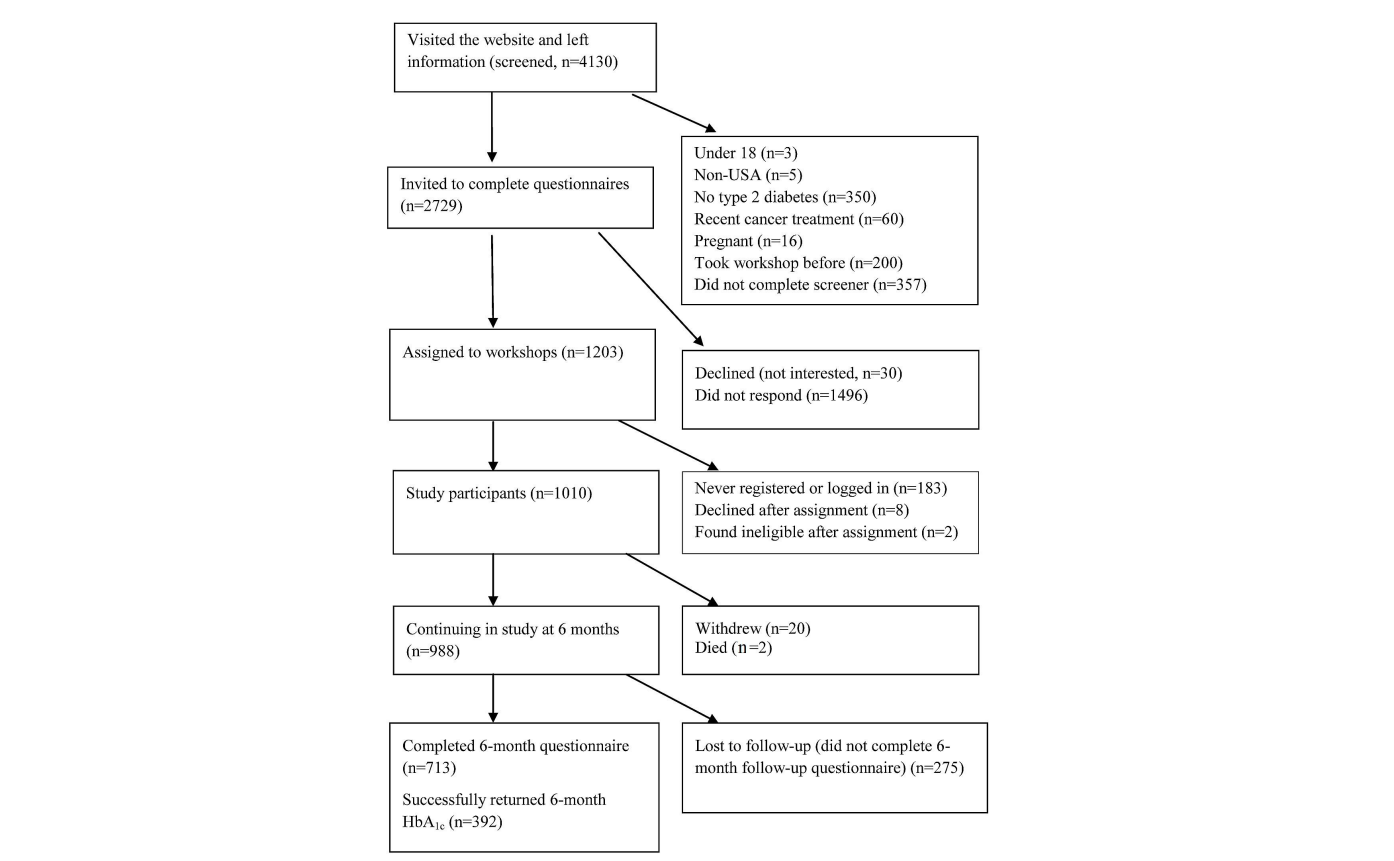
Internet group flowchart. HbA_1c_: hemoglobin A_ic_.

**Figure 2:**
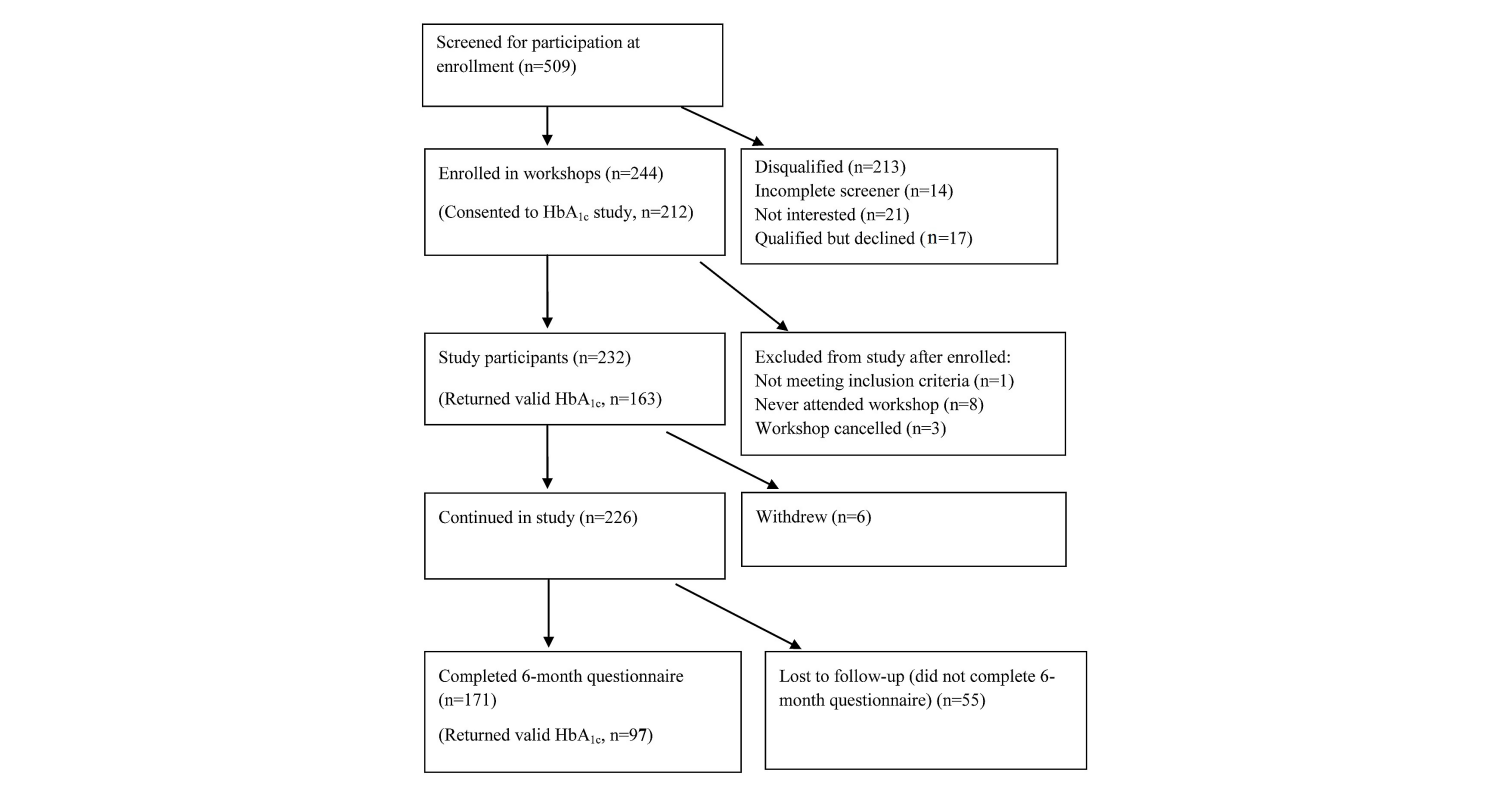
Community workshop group flowchart. HbA_1c_: hemoglobin A_1c_.

There were 4639 potential participants who indicated interest in the study. Of these, 1242 participants met the study criteria, consented, completed baseline questionnaires, and attended at least one session of the workshop, resulting in 1010 Internet participants (Figure 1) and 232 face-to-face participants (Figure 2).

Table 1 gives the demographic characteristics. Participants in the Internet group were statistically more likely to be male (*P*=.02, from *t* test), more likely to be married (*P*<.001), less likely to be a racial/ethnic minority (*P*=.001), to be more educated (*P*=.01), and to be younger (*P*<.001). Overall, the participants tended to be well-educated women.

**Table 1:** Demographic characteristics of the study sample.

Variable		Community (n=232)	Internet (n=1010)	Entire sample (n=1242)
Male, n (%)		61 (26.3%)	346 (34.3%)	407 (32.8%)
Education in years, mean (SD, range)		15.0 (2.86, 8–23)	15.5 (2.75, 9–23)	15.4 (2.78, 8–23)
Married, n (%)		117 (50.4%)	762 (75.5%)	879 (70.8%)
**Ethnicity/race, n (%)**				
	Non-Hispanic white	148 (63.8%)	756 (75.0%)	904 (72.9%)
	Black	70 (30.2%)	98 (9.7%)	168 (13.5%)
	Hispanic	5 (2.2%)	90 (8.9%)	95 (7.7%)
Age in years, mean (SD, range)		65.6 (10.1, 28–95)	55.0 (8.68, 25–91)	57.0 (9.86, 25–95)
Number of other chronic conditions, mean (SD, range)		1.65 (1.37, 0–8)	1.40 (1.15, 0–6)	1.45 (1.19, 0–8)
**Health care coverage, n (%)**				
	Medicare	133 (57.3%)	62 (6.1%)	195 (15.7%)
	Private insurance	166 (71.6%)	1010 (100%)	1176 (94.7%)

### Noncompleters of 6-Month Questionnaires

Outside of HbA_1c_, which was optional, no variable had more than 1.7% missing data among those who completed questionnaires. We did not attempt to impute missing data.

Of the 1242 participants, 358 (28.82%) did not complete the 6-month questionnaire, including 28 who withdrew from the study or died before 6 months (Figure 1,Figure 2). The study retention rate was similar and nonsignificant by type of program: 713/1010 (70.59%) of the Internet and 171/232 (73.7%) of the community participants.

Comparing 6-month noncompleters and completers on demographic variables, those who did not complete the 6-month follow-up tended to be younger (mean 55.4 vs 57.6 years old, *P*<.001). The groups did not differ by sex, marital status, education, or ethnicity/race. Among 17 baseline outcome variables, 1 had statistically significance differences. Noncompleters reported less medication adherence (1.22 vs 1.06 on a 5-point scale, *P*=.02).

### 6-Month Changes (Hypotheses 1–4)

Table 2 shows baseline and 6-month change scores for all study participants. It should be noted that at baseline the mean HbA_1c_ was just under 8 with a standard deviation of 1.4. This suggests that, as predicted, the group was very heterogeneous and that many participants had a low HbA_1c_ at baseline.

**Table 2:** Baseline and 6-month scores for participants with 6-month data in the Internet and workshop groups combined.

Variables		Possible range of values	Desirable score	No.	Baseline		6-Month change		Effect size of change	*P*>| *t* | for change
					Mean	SD	Mean	95% CI		
Adjusted HbA_1c_^a,b^			Lower	489	7.95	1.38	–0.151	–2.47 to 10.06	0.109	.002
PHQ-8^c^ depression		0–24	Lower	877	5.97	5.02	–1.02	–1.30 to –0.74	0.203	<.001
General health		0–5	Lower	884	2.90	0.764	–0.117	–0.163 to –0.071	0.153	<.001
Illness intrusiveness		1–7	Lower	877	2.79	1.20	–0.082	–0.153 to –0.005	0.068	.03
Hypoglycemic symptoms		0–7	Lower	865	1.36	1.40	–0.188	–2.78 to –0.108	0.134	<.001
Fatigue		1–10	Lower	884	4.84	2.34	–0.508	–0.657 to –0.341	0.217	<.001
Sleep		1–10	Lower	882	3.93	2.95	–0.061	–0.240 to 0.119	0.021	.51
Aerobic exercise (min/week)			Higher	876	81.6	101	8.91	2.39 to 15.4	0.088	.01
Medication adherence		0–4	Lower	878	1.05	1.08	–0.135	–0.201 to –0.073	0.125	<.001
Communication with doctor		0–5	Higher	877	2.78	1.17	0.221	0.154 to 0.283	0.189	<.001
**Proportion receiving recommended test in last 6 months^d^**										
	Eye exam	0,1	Higher	877	0.451	0.498	0.100	0.057 to 0.145	0.201	<.001
	Foot exam	0,1	Higher	877	0.551	0.498	0.132	0.096 to 0.166	0.265	<.001
	Cholesterol exam	0,1	Higher	877	0.763	0.425	0.090	0.061 to 0.127	0.222	<.001
	Kidney exam	0,1	Higher	877	0.614	0.487	0.152	0.115 to 0.190	0.312	<.001

^a^HbA_1c_: hemoglobin A_1c_.

^b^Because of a laboratory recalibration changing HbA_1c_ measurement, HbA_1c_ prior to June 2014 was adjusted by adding 0.4.

^c^PHQ-8: 8-item Patient Health Questionnaire.

^d^“Don’t know” set to “no examination.”

Most health status and behavior variables showed statistically significant 6-month improvements. This remained when adjusting for multiple comparisons (using a *P* value of .003 as the criterion for significance).

For intent-to-treat analyses (not shown), we substituted a value of 0 (no change) for 6-month change scores for those who did not complete 6-month questionnaires. The intent-to-treat analyses of changes resulted in no differences from the *P* values shown in Table 2.

For all subsequent analyses, we included only those who completed 6-month questionnaires. We present these analyses as a means of determining who benefited in this heterogeneous population.

### Increase in Receiving Recommended Tests (Hypothesis 4)

Of those who had not been examined (tested) in any of the 4 areas (eye, foot, cholesterol, kidney) in the year before baseline (n=85), only 12 (14%) had no tests in the next 6 months (not shown in tables). Thus, 73 (86%) of those who had not been examined at all had at least one examination in the 6 months after the program. A total of 46 (54%) of these had 3 or more of the 4 tests.

### Differences in Changes by Type of Program or Location (Hypothesis 5)

There was little evidence of differences between participants in the two types of programs (Internet vs community). Only 1 change score differed by program: community participants had greater improvement in sleep (*P*<.001). Similarly, there was little evidence of differences by location of community programs. The only difference by community was for improvements in foot examinations, where community participants in St. Louis had marginally less improvement than in the other 2 communities (*P*=.04).

### Analyses of Specific End Points (Hypothesis 6)

For each of the 5 outcomes described in hypothesis 6 above, we look at 6-month changes for those who exhibited symptoms and those who did not (not shown in the tables). Then we looked at those who benefited for 1 or more of these 5 problems.

#### HbA1c ≥9

Of those who supplied a blood sample for HbA_1c_ determination at both baseline and 6 months, 20.0% (98/489) had a baseline HbA_1c_ of ≥9. The proportion with HbA_1c_ >9 was reduced to 15.3% at 6 months (n=74, *P*<.001 from chi-square). The mean reduction in HbA_1c_ for the group starting at ≥9 was –0.93 (*P*<.001, effect size 0.73). For those with <9 at baseline, there was an increase of 0.05 in HbA_1c_ (*P*=.26).

#### Depression

At baseline, 22.0% (193/877) had symptoms indicating clinical depression (PHQ-8 score of ≥10) [24]. This was reduced to 16.3% at 6 months (n=144, *P*<.001). Baseline mean PHQ for the “depressed” group was 13.8 (SD 3.34). At 6 months, the mean reduction was 3.87 (*P*<.001, effect size 1.1); and 51.3% of those originally with a score ≥10 (n=99) had scores of <10. For those who had a score of <10 at baseline, there was a mean reduction of –0.21 in PHQ scores (*P*=.08)

#### Hypoglycemia

At baseline 332/865 (38.4%) had 2 or more symptoms of hypoglycemia (mean 2.88, SD 1.04). At 6 months, the number with 2 or more symptoms was reduced to 280 (32.4%). The 6-month change in mean hypoglycemic symptoms was –0.91 for those who had 2 or more symptoms at baseline (*P*<.001) and was an increase of 0.25 for those who had fewer than 2 symptoms at baseline (*P*<.001).

#### Medication-Taking Adherence

At baseline, 35.0% (n=307) were nonadherent in taking medicine (≥2 on a 0–4 scale), and at 6 months, 29.4% (n=260) were nonadherent (*P*<.001). For participants who were nonadherent at baseline, adherence improved by a mean of 0.73 (SD 1.05, *P*<.001, effect size 0.59) at 6 months, and 124 (40.4%) of the previous nonadherent participants were considered adherent. For those who had been adherent at baseline (n=575), there was an increase of 0.18 in mean nonadherence (*P*<.001).

#### Exercise

At baseline, 23.2% (203/876) indicated that they were taking no aerobic exercise. This was reduced to 18.4% (n=163) at 6 months (*P*<.001). At 6 months, the mean increase in exercise for nonexercisers was 43 minutes (SD 73, *P*<.001) of exercise per week, and 124 (61.1%) of the nonexercisers reported some aerobic exercise. For those reporting some exercise, there was a mean decrease of 1.4 minutes per week (*P*=.61).

In each of the cases above, the improvements for the less well-off group (high HbA_1c_, indications of depression, etc) were much greater than any negative changes for those who had been doing well at baseline.

#### Percentage of Participants With at Least One Problem

At baseline, 70.4% (622/884) of participants fell into 1 or more of the above groups (high HbA_1c_, high depression, hypoglycemic, nonadherent, or nonexercisers); 40.6% had 2 or more problem scores and 19.5% had 3 or more. The 5 criteria variables were not correlated with each other, with the exception of hypoglycemic symptoms and PHQ depression (*r*=.37).

#### Percentage of Participants Benefiting

We then looked at what proportion of the total study population completing 6-month questionnaires improved in at least one of the 5 criteria variables (hypothesis 7). Using effect-size improvements of at least 0.4 as an indication of an improvement, 75.0% of the study population (n=662) improved in at least one of the 5 criteria variables, and 37.1% (n=327) improved in 2 or more. The mean number of improvements (of ≥0.4 effect size) was 1.13. When broken down by how many of the 5 of these problems a participant had, the mean number of improvements tended to increase with the mean number of problems. Those with none of the 5 criteria problems had a mean of 0.671 improvements (out of a possible 5, n=268); those with 1 problem or condition had a mean of 1.08 improvements (n=272); those with 2 problems had 1.31 improvements (n=195); those with 3 problems had 1.52 improvements (n=125); and those with 4 or 5 problems had 2.06 improvements (n=52). There was a correlation (Pearson *r*=.35) between the number of problems and the number of improvements (*P*<.001).

## Discussion

There were modest but statistically significant improvement in 13 of the 14 outcome measures, and 10 of those were significant at the *P*<.001 level. There were significant increases in those completing suggested laboratory tests for diabetes. There was no significant change in quality of sleep. This consistency and level of significance suggests that these improvements were not the result of multiple testing. If we apply a Bonferroni correction and use .003 as the level of significance, 11 of 17 outcomes remain statistically significant.

BCBH-D may help meet the Healthy People 2020 diabetes objectives. The proportion of study participants with HbA_1c_ >9 was reduced from 20% (n=98) to 15% (n=74), and the proportion of participants tested for foot and microalbumin (kidney function) in the last 6 months increased by 13% and 15% (Table 2), respectively. This program also adds to the number receiving formal diabetes education.

Because this is a translation study, participants were not screened for HbA_1c_ or other symptoms. This led to a high degree of heterogeneity among study participants. In addition, many participants were already managing their diabetes and had little room for improvement. It was important, therefore, to determine who might benefit. It appears that those who had an HbA_1c_ of ≥9 decreased their HbA_1c_ by approximately the same amount as one would expect by taking metformin [30]. In addition, 75.0% (n=662) of the sample improved by an effect size of 0.4 or more for at least one of HbA_1c_, depression, hypoglycemia, adherence to medications, and minutes of exercise. Those with none of the 5 problems still tended to benefit, with a mean improvement in 0.67 out of 5 possible criterial outcomes. However, as might be expected, those with more “problems” had more benefits (ie, making a greater mean number of improvements of ≥0.4 effect size among the 5 criterial measures).

### 6-Month Noncompleters

Considering the large initial sample (N=1242) and large number of outcomes, there were few statistical differences between 6-month completers and noncompleters. Based on the significant difference in baseline medication adherence, there is some evidence that noncompleters were slightly more likely to be medication noncompliers. They were also slightly younger. There is no evidence that they were more ill or had greater severity of symptoms, which would suggest that attrition would have been unlikely to bias 6-month outcomes.

### Modes of Delivery

Despite a few differences at baseline, there were few differences in 6-month changes between those attending community workshops and the Internet group. This suggests that both modes are similarly effective. In this study, very few people had a choice of modes. However, it may be that future studies will find a greater population penetration when people are offered a choice.

### Limitations

Because we lack a control group, we cannot be certain that the improvements observed in this study are not due to other factors. Plausible alternative explanations for the improvements include the introduction of new medications in the marketplace and health plan initiatives that were implemented for the purpose of improving the health of individuals with chronic conditions. It is also possible that there was a beneficial effect from participating in the workshops resulting in a greater likelihood of participants taking advantage of the Anthem initiatives and new medications. While these other factors are important to keep in mind, the consistency of the statistically significant improvements across multiple domains suggests a favorable impact of workshop participation.

By looking at the participants who were worse off on specific measures, we risk that part of the observed improvements might have been the result of regression to the mean. While regression to the mean may have contributed to subgroup improvements, it most likely does not explain all of the improvement.

Most of this study population were covered by a health plan and had a high mean baseline education (15.4 years). This may have contributed to the positive results. However, there were similar results when the small-group program was used with a low-education (mean of 7 years) Spanish-speaking population [8].

Data on how well improvements are sustained after 6 months are not included in this study. A future study will examine 12-month outcomes and address the sustainability of improvements following the intervention.

In order to reduce participant burden, unfortunately we did not include self-efficacy to manage diabetes in the questionnaires for this study. The program was designed to enhance self-efficacy and has been found to be significantly associated with self-efficacy in earlier randomized trials [7-9]. In a future translation study, it would be desirable to include a measure of self-efficacy to manage diabetes [31].

We did not attempt to control for changes in medication usage. Choosing to take medication for diabetes may be a part of self-management, and thus adding medication could be a positive outcome. In other cases, participants may have begun taking medication because of worsening health. A future study might want to look carefully at the relationships between self-management education, medication change, and health outcomes, as well as at other changes in behaviors as mediating variables.

### Conclusion

As a community-based public health intervention, BCBH-D offered in two modes demonstrated small but significant benefits. It also showed promise for helping to meet at least some of the Healthy People 2020 diabetes objectives. More important, it demonstrated clinically significant benefits for those with high HbA_1c_, and important benefits for those with depression and hypoglycemia, as well as nonadherers to medication and nonexercisers. The benefits differed by individual, but a large majority of the population demonstrated meaningful improvements in at least one of the above areas. This study demonstrated that the peer-facilitated BCBH-D in both face-to-face and Internet formats can assist a national sample of health plan members in improving their diabetes management.

## Abbreviations

BCBH-D: Better Choices, Better Health-Diabetes
HbA1c: hemoglobin A1c
PHQ-8: 8-item Patient Health Questionnaire
